# Single-cell data revealed exhaustion of characteristic NK cell subpopulations and T cell subpopulations in hepatocellular carcinoma

**DOI:** 10.18632/aging.205723

**Published:** 2024-04-05

**Authors:** Zhongfeng Cui, Hongzhi Li, Chunli Liu, Juan Wang, Chunguang Chen, Shanlei Hu, Xiaoli Zhao, Guangming Li

**Affiliations:** 1 Department of Clinical Laboratory, Henan Provincial Infectious Disease Hospital, Zhengzhou 450000, China; 2 Department of Tuberculosis, Henan Provincial Infectious Disease Hospital, Zhengzhou 450000, China; 3 Department of Infectious Diseases and Hepatology, Henan Provincial Infectious Disease Hospital, Zhengzhou 450000, China

**Keywords:** hepatocellular carcinoma, immunotherapy, exhausted T cells, exhausted NK cells, transcription factor network

## Abstract

Background: The treatment and prognosis of patients with advanced hepatocellular carcinoma (HCC) have been a major medical challenge. Unraveling the landscape of tumor immune infiltrating cells (TIICs) in the immune microenvironment of HCC is of great significance to probe the molecular mechanisms.

Methods: Based on single-cell data of HCC, the cell landscape was revealed from the perspective of TIICs. Special cell subpopulations were determined by the expression levels of marker genes. Differential expression analysis was conducted. The activity of each subpopulation was determined based on the highly expressed genes. CTLA4+ T-cell subpopulations affecting the prognosis of HCC were determined based on survival analysis. A single-cell regulatory network inference and clustering analysis was also performed to determine the transcription factor regulatory networks in the CTLA4+ T cell subpopulations.

Results: 10 cell types were identified and NK cells and T cells showed high abundance in tumor tissues. Two NK cells subpopulations were present, FGFBP2+ NK cells, B3GNT7+ NK cells. Four T cells subpopulations were present, LAG3+ T cells, CTLA4+ T cells, RCAN3+ T cells, and HPGDS+ Th2 cells. FGFBP2+ NK cells, and CTLA4+ T cells were the exhaustive subpopulation. High CTLA4+ T cells contributed to poor prognostic outcomes and promoted tumor progression. Finally, a network of transcription factors regulated by NR3C1, STAT1, and STAT3, which were activated, was present in CTLA4+ T cells.

Conclusion: CTLA4+ T cell subsets in HCC exhibited functional exhaustion characteristics that probably inhibited T cell function through a transcription factor network dominated by NR3C1, STAT1, and STAT3.

## INTRODUCTION

Liver cancer is the third leading cause of death due to cancer, with approximately 820,000 deaths annually [1]. Hepatocellular carcinoma (HCC) is the most common primary liver cancer. There are significant geographic differences in the incidence and mortality of hepatocellular carcinoma (HCC) worldwide, especially between Eastern (China, Japan, and Korea) and Western (e.g., the United States and European countries) countries. These differences are mainly attributed to differences in etiology, risk factors, genetic background, and prevention and treatment strategies. In Eastern countries, the main risk factors for HCC are hepatitis B and C virus (HBV and HCV) infection, and aflatoxin exposure. In contrast, in Western countries, the rising trend in HCC is mainly associated with an increase in non-alcoholic fatty liver disease (NAFLD) and metabolic syndrome, although HBV and HCV infections remain important risk factors. [2–5]. As an extremely serious public health problem, the development of therapeutic options for HCC has been the subject of attention. At this stage, HCC is mainly treated with surgery, radiotherapy, and chemotherapy, but these treatments are often effective only at the initial stage of HCC treatment, and eventually most cases become resistant to these traditional treatments and eventually develop extensive metastasis [6]. The early clinical symptoms of HCC are insidious, and the site of disease is often close to other vital organs of the body, which exacerbates the difficulty of traditional diagnosis and treatment [7]. Recently, the development of immunotherapy has raised expectations for the treatment of advanced HCC around the world [8]. The immunotherapeutic approach of the PD-L1 inhibitor Atezolizumab combined with the monoclonal antibody bevacizumab, which suppressed cancer migration, was effective in the treatment of advanced HCC [9]. However, the lack of effective therapeutic targets and limited response rate to the disease were always the general problems of immunotherapy, especially immune checkpoint inhibition therapy [10, 11]. Over the years, basic research for HCC has not led to breakthroughs in treatment and prognosis, and there is a requirement to approach the search for specific therapeutic agents and methods from a new perspective. This relies on a comprehensive understanding of the molecular mechanisms underlying the development of the disease, and therefore, unraveling the molecular mechanisms underlying the biological behavior of HCC is crucial for the prevention and treatment of HCC.

The rapid development of immunotherapy has deepened insights into cancer, and studies have concluded that the tumor microenvironment (TME) is an important factor influencing immunotherapy [12]. The function of tumor-infiltrating immune cells in the TME is critical for regulating immunotherapy. In immunotherapy, T cells and NK cells are two key immune cells that play a crucial role in recognizing and destroying cancer cells [13]. T cells are part of the adaptive immune system and are capable of recognizing and responding specifically to specific antigens. In par therapy, T cells initiate an immune response against tumor cells by recognizing and binding to specific antigens on the surface of these cells through their T cell receptor (TCR). Once activated, CD8+ T cells (also known as cytotoxic T cells) can directly kill tumor cells. In addition, T cells are able to form memory cells that provide long-term immune protection against tumor recurrence [14]. Natural killer cells (NK cells) are an important type of immune cells. NK cells are the source of pro-inflammatory cytokines and chemokines in TME, which activate T cells or other immune cells (e.g., macrophages) to achieve an adaptive immune response [15]. However, when NK cells and T cells are functionally depleted, the organism is unable to achieve an anti-tumor response, and this exacerbates cancer progression [16, 17]. T cells and NK cells can interact and synergize in the immune response. T cells enhance NK cell activity by secreting the cytokine, IFN-γ, and T cells can enhance NK cell activity. In immunotherapies such as immune checkpoint inhibitors, CAR-T cell therapy, and NK cell-based therapies, the roles of T cells and NK cells are used to enhance the immune response to cancer. T cells and NK cells play complementary and mutually reinforcing roles in immunotherapy, and their interactions are critical for designing more effective cancer treatment strategies [18]. Improving NK cells and T cells exhaustion in cancer patients is essential to enhance survival.

In this study, we obtained single-cell RNA sequencing data of hepatocellular carcinoma (HCC) and healthy liver tissues from the GEO database. Data preprocessing and screening were performed by Seurat package to retain eligible cells, and PCA and UMAP were used to perform downscaling and clustering analyses to identify different cell subpopulations. Differential expression analysis was used to explore gene expression heterogeneity among cell subpopulations. Biological pathways involved in cellular subpopulations were identified by single sample enrichment analysis (ssGEEA). Further, SCENIC analysis was used to reveal the transcription factor networks within CTLA4+ T cell subpopulations. In addition, transcriptome sequencing analysis and survival analysis were performed to assess the clinical relevance of cell subpopulations through the TCGA database.

## MATERIALS AND METHODS

### Analysis of HCC single-cell data

The single-cell sequencing (scRNA-seq) data of tissue from HCC and healthy liver samples were collected from the Gene Expression Omnibus (GEO, https://www.ncbi.nlm.nih.gov/geo/) database. The download registration number was GSE162616, which contained three HCC samples and three normal samples. For analyzing the scRNA-seq data, the Seurat package [19] was loaded from the R programming software. The Read10X function was first called to read the scRNA-seq data and retain the valid sequencing data. Cells satisfying gene number between 200 and 3000 and mitochondrial gene proportion <10% were retained. The NormalizeData function was loaded to conduct the log transformation. To determine the highly variable genes (HVGs) among them, the FindVariableFeatures function was loaded to determine the top 2,000 HVGs based on the relationship between the mean and variance of expression, and principal component analysis (PCA) was performed using these HVGs. Before principal component analysis, we called the ScaleData function to normalize the expression values of all genes. After PCA downscaling, the batch effect between the six samples was handled by the harmony package [20] The analysis parameters were set to max.iter.harmony = 50, lambda = 0.5, assay.use = “SCT”. Uniform Manifold Approximation and Projection (UMAP) dimensionality reduction analysis was conducted based on the first 30 principal components in the PCA, and finally the cells were clustered into clusters by the FindNeighbors and FindClusters functions.

### Cellular annotation

The marker genes of the cells were obtained from the CellMarker database (http://xteam.xbio.top/CellMarker/), and the cell types previously clustered into clusters were annotated and analyzed based on the expression levels of the marker genes. For the identified NK cells and T cells, the parameter resolution = 0.1 was set for further cellular annotation.

### Differential expression analysis between cell subpopulations

To explore the heterogeneity of gene expression patterns between identified cell subpopulations, the FindAllMarkers function from the Seurat package was called to calculate the highly expressed genes for each cell subpopulation. The parameters were set to: only.pos = T, min.pct = 0.25, logfc.threshold = 0.25.

### HCC transcriptome sequencing analysis

HTSeq-FPKM data of TCGA-Liver Cancer (LIHC) with expression value of log2 (fpkm+1) were downloaded from University of California, Santa Cruz (UCSC) Xena website (https://xena.ucsc.edu/). We also obtained the overall survival and survival status of the samples in TCGA-LIHC. The data from TCGA-LIHC were imported into the Sangerbox database (http://sangerbox.com/). The Sangerbox is an online biometrics analysis database site that provides a wealth of analytical tools for online statistics on patients' clinical information [21]. Ensembl IDs were converted to gene symbols according to the gencode.v22.annotation.gene.probeMap file obtained from UCSC Xena.

### Enrichment analysis and survival analysis and gene set enrichment analysis

For the identified subpopulations of NK cells and T cells and the highly expressed genes therein, the GSVA package [22] was invoked to conduct a single-sample gene set enrichment analysis (ssGSEA) to determine the enrichment scores for each cell subpopulation. The median value of the enrichment scores of the cell subpopulations was used to group the samples in TCGA-LIHC into high-enrichment score groups and low-enrichment score groups. Differences in survival between the two groups were assessed by Kaplan-Meier (K-M) curves, and the statistic of choice was the log-rank test. Fifty Hallmark gene sets were extracted from the Molecular Signatures Database (MSigDB, https://www.gsea-msigdb.org/gsea/msigdb). We calculated the ssGSEA enrichment scores for the hallmark gene sets of the samples in TCGA-LIHC.

### Single-cell regulatory network inference and clustering analysis

Single-cell regulatory network inference and clustering (SCENIC) [23] is an algorithm for gene regulatory network (GRNs) developed specifically for single-cell data. Its innovation is the introduction of gene co-expression networks inferred from transcription factor motif sequence validation statistical methods to identify highly reliable transcription factor-dominated GRNs. We used the GENIE3 method to calculate the potential target genes of each transcription factor (TF) and top5perTarget to construct the transcription factor regulatory network according to the official tutorial of SCENIC (http://scenic.aertslab.org/). Eventually we identified highly plausible TF-target gene relationship pairs and used the AUCell function to calculate the degree of regulon activity in each cell. Each regulon is a gene set of transcription factors and their directly regulated target genes, and the SCENIC package would score the activity of each regulon in individual cells. The scoring is based on the expression value of the gene, with higher scores representing greater activation of the gene set.

### Statistical analysis

The Wilcoxon rank-sum test was used to compare the differences in continuous variables between two groups, and the Kruskal-Wallis test was used to compare the differences between continuous variables in more than three groups. For survival analysis, we divided TCGA-LIHC patients into high and low groups based on the median of continuous variables, and then used the log-rank test to compare the differences in survival time between the two groups. The Pearson correlation was used to measure the correlation between CTLA4+ T-cell subsets and hallmark enrichment scores. All calculations were completed by the R language (version 4.3.1). For all statistical analyses, *p* < 0.05 was considered statistically significant.

### Data availability statement

The datasets generated during and analyzed during the current study are available from the corresponding author on reasonable request.

## RESULTS

### Cell landscape of TIICs in HCC

**Figure 1:**
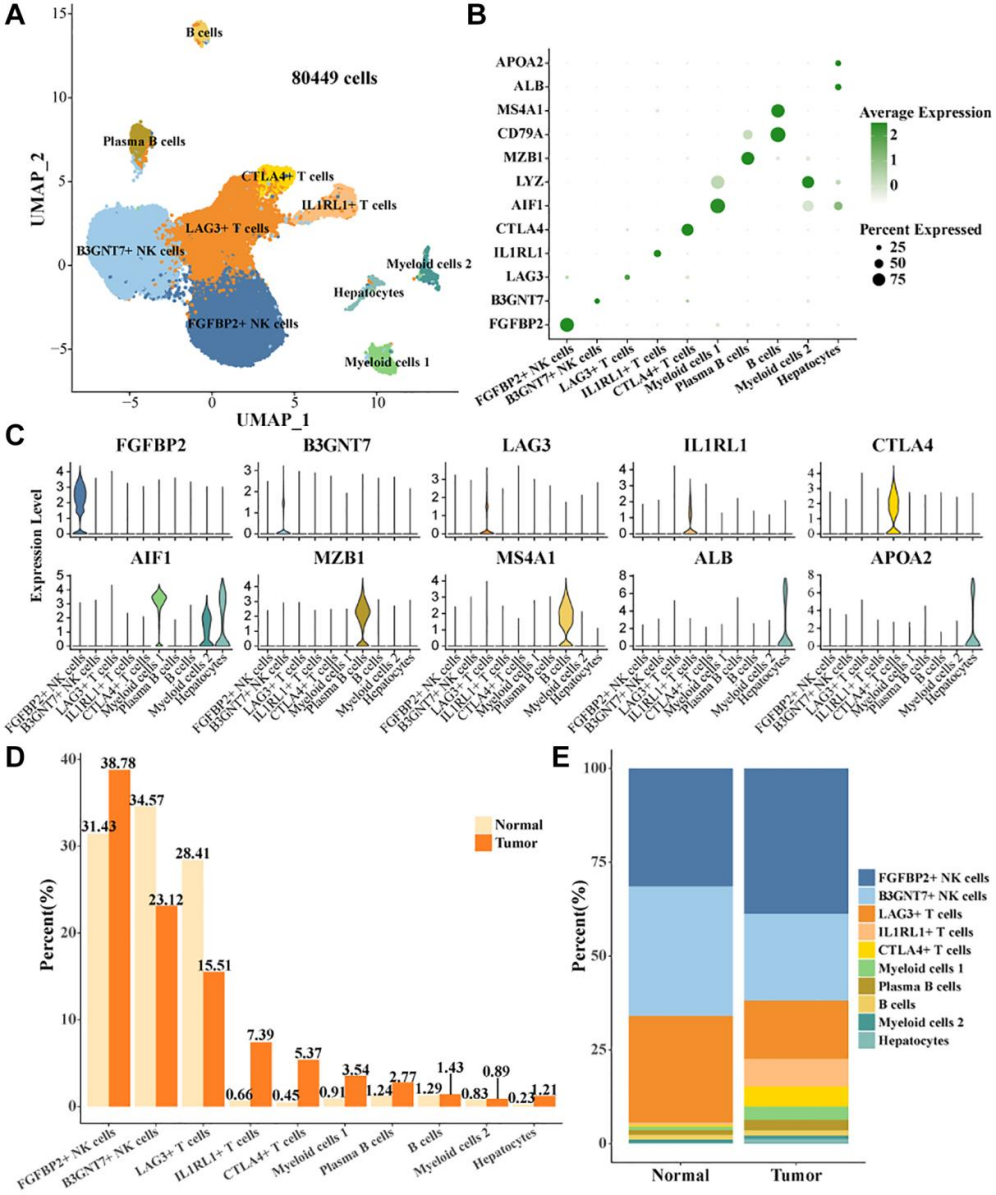
**Cell landscape of TIICs in HCC.** (**A**) Distribution of 10 cell types. (**B**) Bubble plots demonstrate the expression levels of marker genes in 10 cell types. (**C**) Violin plot of marker genes expression levels in 10 cell types. (**D**, **E**) Proportion statistics of 10 cell types in tumor tissues and normal tissues.

Single-cell data in GSE162616 were filtered, normalized, batch utility removed, downscaled, and clustered by the Seurat package, and 80,449 high-quality cells were retained (Supplementary Figure 1A–1D). Cell clusters were annotated based on expression levels of marker genes (Figure 1A, 1B), 10 cell types were identified, B cells, Plasma B cells, CTLA4+ T cells, IL1RL1+ T cells, LAG3+ T cells, B3GNT7+ NK cells, Myeloid cells 2, Hepatocytes, FGFBP2+ NK cells, Myeloid cells 1 (Figure 1C). According to the statistical results, the proportions of FGFBP2+ NK cells, IL1RL1+ T cells, CTLA4+ T cells, Myeloid cells 1, and Plasma B cells were higher than that of normal in tumor tissues. The proportions of B3GNT7+ NK cells, LAG3+ T cells were higher in Normal tissues (Figure 1D, 1E).

### Subpopulations of cells characterized by NK cells and T cells depletion are present in HCC tissues

**Figure 2:**
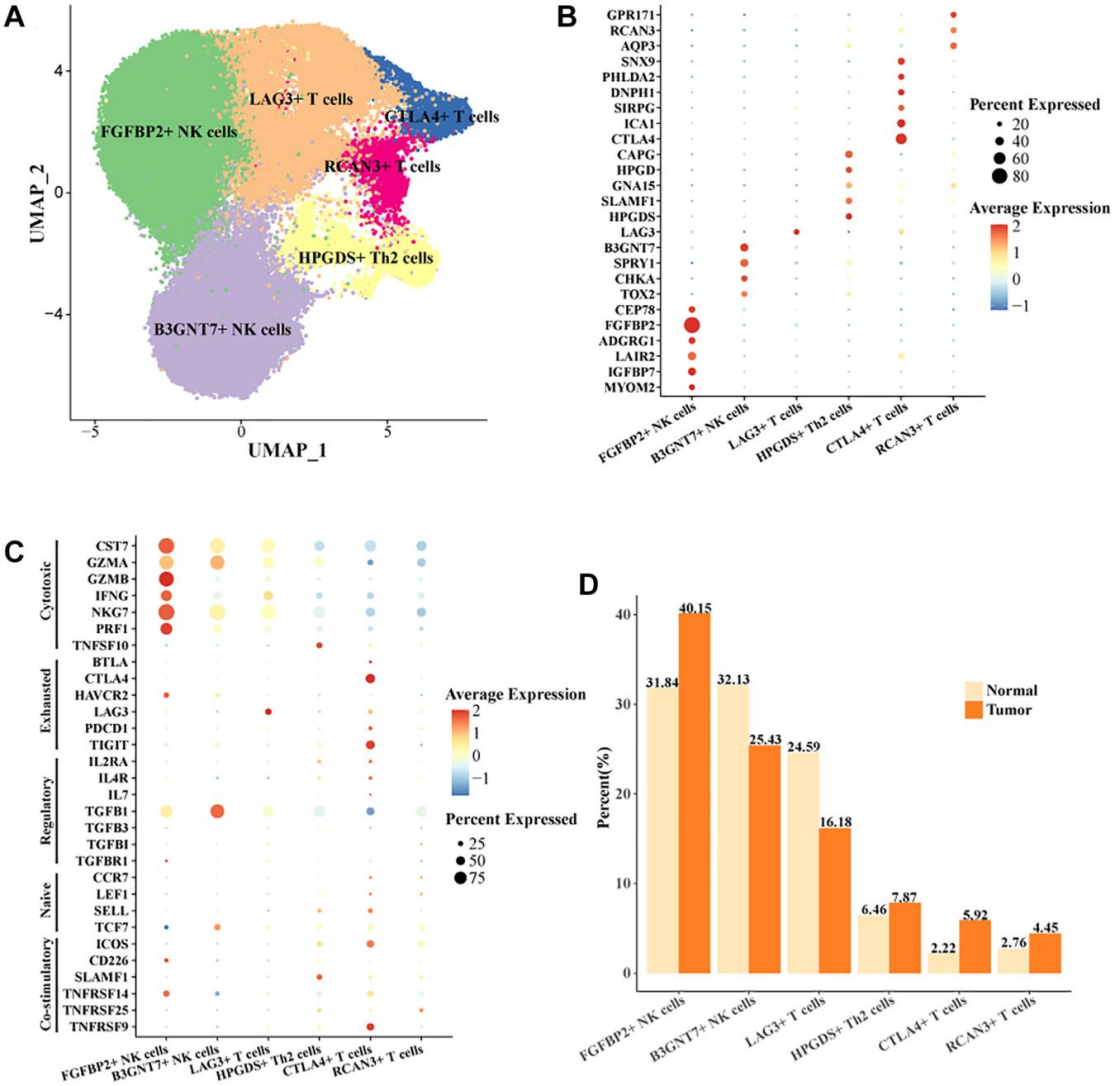
**Cell landscape of exhausted FGFBP2+ NK cells, CTLA4+ T cells in HCC.** (**A**) Distribution of 6 cell subtypes in NK cells and T cells. (**B**, **C**) Expression levels of marker genes in the 6 cell subtypes. (**D**) Proportion of the 6 cell subtypes in tumor tissues and normal tissues.

Studies have shown that the function of NK cells and T cells plays a key role in the treatment of HCC [24]. Studies also showed that the function of T cells in HCC was a critical factor in regulating immunotherapy [25]. To reveal the mode of action of NK cells and T cells in HCC, we extracted all NK cells and T cells in the cell landscape for further cellular annotation. We noted the presence of two cell subtypes in NK cells, FGFBP2+ NK cells, B3GNT7+ NK cells. Four cell subtypes were present in T cells, LAG3+ T cells, CTLA4+ T cells, RCAN3+ T cells, HPGDS+ Th2 cells (Figure 2A). The expression levels of the marker gene in the six cell subtypes were demonstrated in Figure 2B, 2C. CTLA4 was the key stimulatory receptor during T cell activation, and CTLA4 modulated multiple autoimmune disease responses [26]. FGFBP2 was the signature gene for cytotoxic killing function in NK cells [27]. As demonstrated by our results, FGFBP2+ NK cells expressed the highest level of killing-related genes, suggesting that it might play a tumor cell killing role in the immune microenvironment. Meanwhile, HAVCR2 and TGFB1 were overactivated in FGFBP2+ NK cells. The expression level of HAVCR2 was higher in tumor tissues. NK cells with high expression of HAVCR2 were in a terminal state with diminished signaling function and metabolic activity [28]. Our results demonstrated that FGFBP2+ NK cells in tumor tissues exhibited a functionally inhibited state due to overexpression of HAVCR2 (Supplementary Figure 2A). B3GNT7+ NK cells highly expressed TGFB1 and TCF7 (Supplementary Figure 2B). TCF7 was a biomarker for naïve or undifferentiated cells [29]. The results suggested that B3GNT7+ NK cells might be naïve cell subtypes. LAG3+ T cells highly expressed LAG3 and CTLA4+ T cells highly expressed CTLA4 and TIGIT, which were also higher in tumor tissues than in normal tissues (Supplementary Figure 2C, 2D). CTLA4 and TIGIT are marker genes for depleted T cells [30]. CTLA4+ T cells may be a subpopulation of T cells characterized by depletion in HCC, with potential correlation to the suppressed state of the immune microenvironment. In addition, we found a higher proportion of FGFBP2+ NK cells, CTLA4+ T cells, and a lower proportion of LAG3+ T cells in tumor tissues compared to normal tissues (Figure 2D). Overall, we identified exhaustive immune cell subpopulations, FGFBP2+ NK cells, CTLA4+ T cells based on the cellular landscape of TIICs in HCC.

### High CTLA4+ T cells portend poor prognosis in HCC

**Figure 3:**
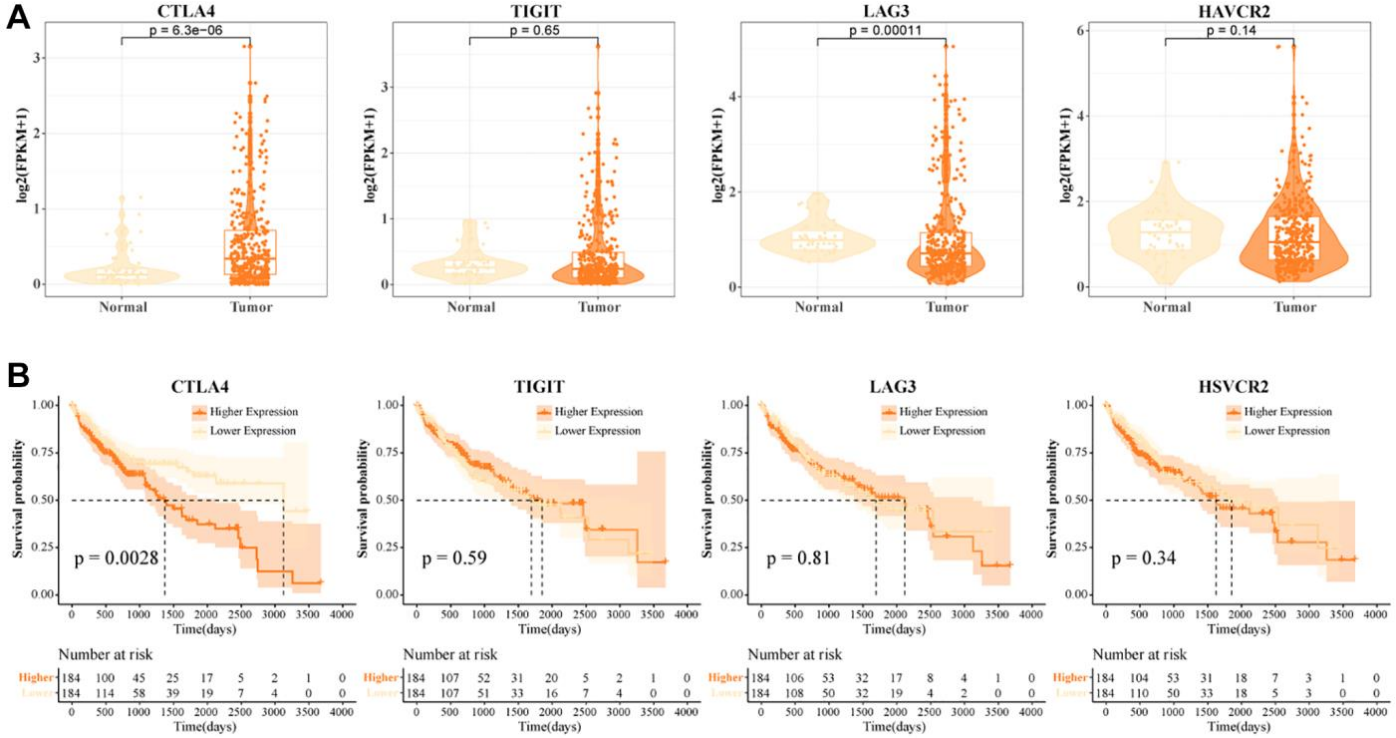
**High CTLA4+ T cells portend a poor prognosis for HCC.** (**A**) Expression levels of CTLA4, TIGIT, LAG3, and HAVCR2 in tumor tissues and normal tissues in TCGA-LIHC data. (**B**) K-M curves of HCC patients in CTLA4, TIGIT, LAG3, and HAVCR2 subgroups in TCGA-LIHC data.

In the TCGA-LIHC data, we examined the expression levels of CTLA4, TIGIT, LAG3, and HAVCR2. We found that CTLA4 was highly expressed in tumor tissues and LAG3 was lowly expressed in tumor tissues (Figure 3A). High levels of CTLA4 might be a critical factor contributing to the immunosuppressive microenvironment in HCC. LAG3 was a promising immune checkpoint, and overexpression of LAG3 promoted tumor cell development by forming an immunosuppressive microenvironment to suppress the activity of immune cells [31]. When T cells were activated by cytokine stimulation, LAG3 was secreted in large quantities on their surface and this suppressed T cell function [32]. A greater percentage of LAG3+ T cells in normal tissues was found in the previous results. Low expression of LAG3 in tumor tissues might promote the tumor killing effect of T cells, but the number of LAG3+ T cells recruited in tumors was not sufficient to support their anti-tumor effect. Further, we found that patients with high expression of CTLA4 exhibited a suboptimal prognostic outcome (Figure 3B). Overall, high abundance of CTLA4+ T cells and high levels of CTLA4 in tumor tissues contributed to the poor prognosis of HCC.

### CTLA4+ T cells promoted HCC progression

**Figure 4:**
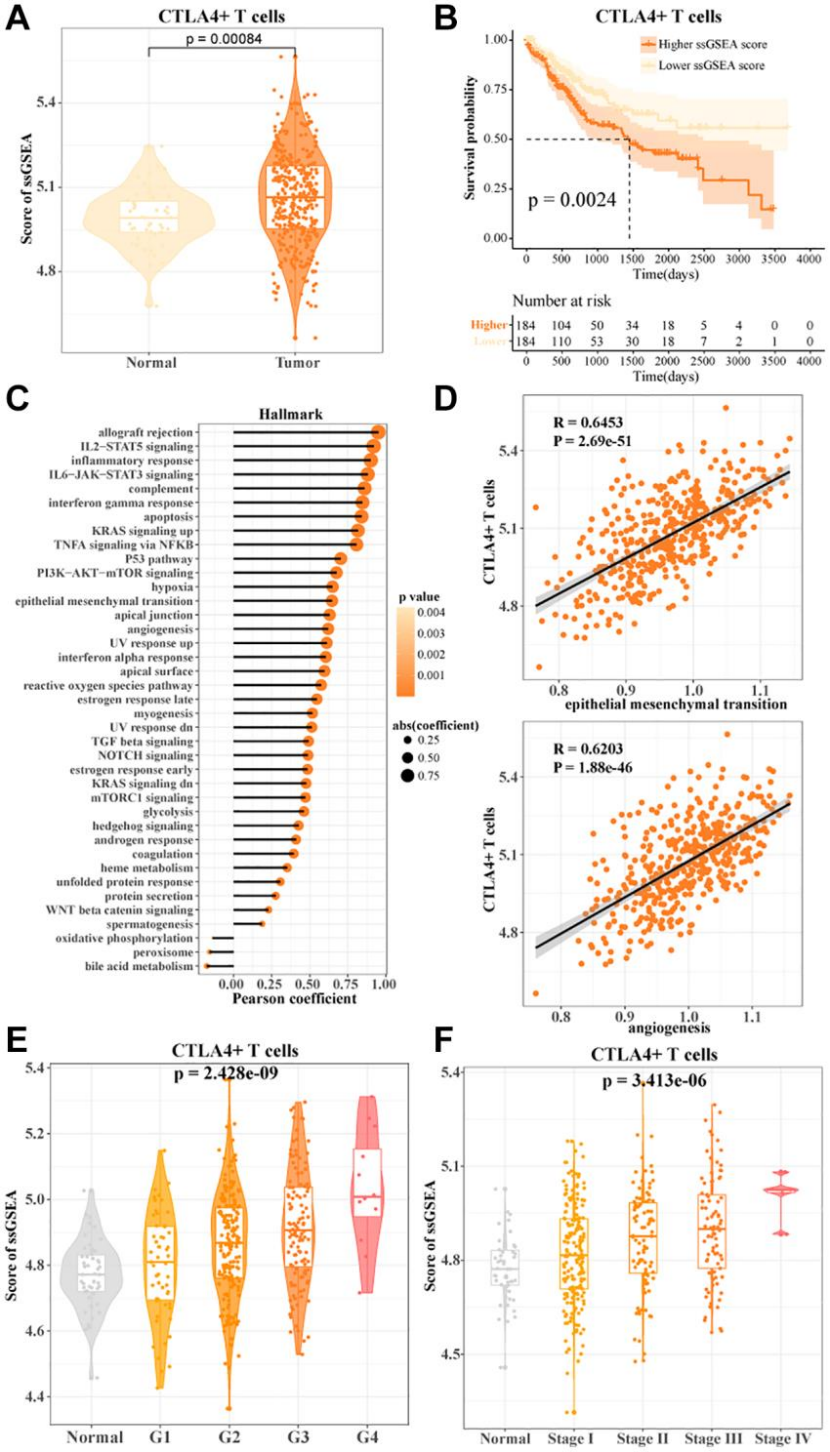
**CTLA4+ T cells promoted HCC progression.** (**A**) The ssGSEA score of CTLA4+ T cells in normal and tumor tissues in TCGA-LIHC data. (**B**) K-M curves of HCC patients in the high/low CTLA4+ T cells scoring groups. (**C**, **D**) Pearson's correlation between CTLA4+ T cells scores and cancer-related pathways. (**E**, **F**) CTLA4+ T cells scores in normal tissue and grade subgroups, stage subgroups.

In the TCGA-LIHC data, we found a higher enrichment score for CTLA4+ T cells (Figure 4A). The more CTLA4+ T cells activity, the worse the prognosis of HCC patients (Figure 4B). Notably, FGFBP2+ T cells, B3GNT7+ T cells, LAG3+ T cells, HPGDS+ T cells, and RCAN3+ T cells activities were not significantly associated with the prognosis of HCC patients (Supplementary Figure 3). CTLA4+ T cells were potential indicators for assessing the prognosis of HCC. To further investigate the correlation between CTLA4+ T cells and cancer-related pathways, we found that CTLA4+ T cells showed significant positive correlations with inflammatory response, angiogenesis, reactive oxygen species pathway, epithelial-to-mesenchymal transition, and P53 pathway (Figure 4C, 4D). We also found that CTLA4+ T cells activity showed an overall positive correlation with clinicopathologic stage. As grade and stage increased, CTLA4+ T cells activity also increased in HCC patients (Figure 4E, 4F). In connection with the results of the previous analysis, our data suggested that CTLA4+ T cells activity might be a pivotal regulator of HCC progression.

### Transcription factor regulatory network in CTLA4+ T cells

**Figure 5:**
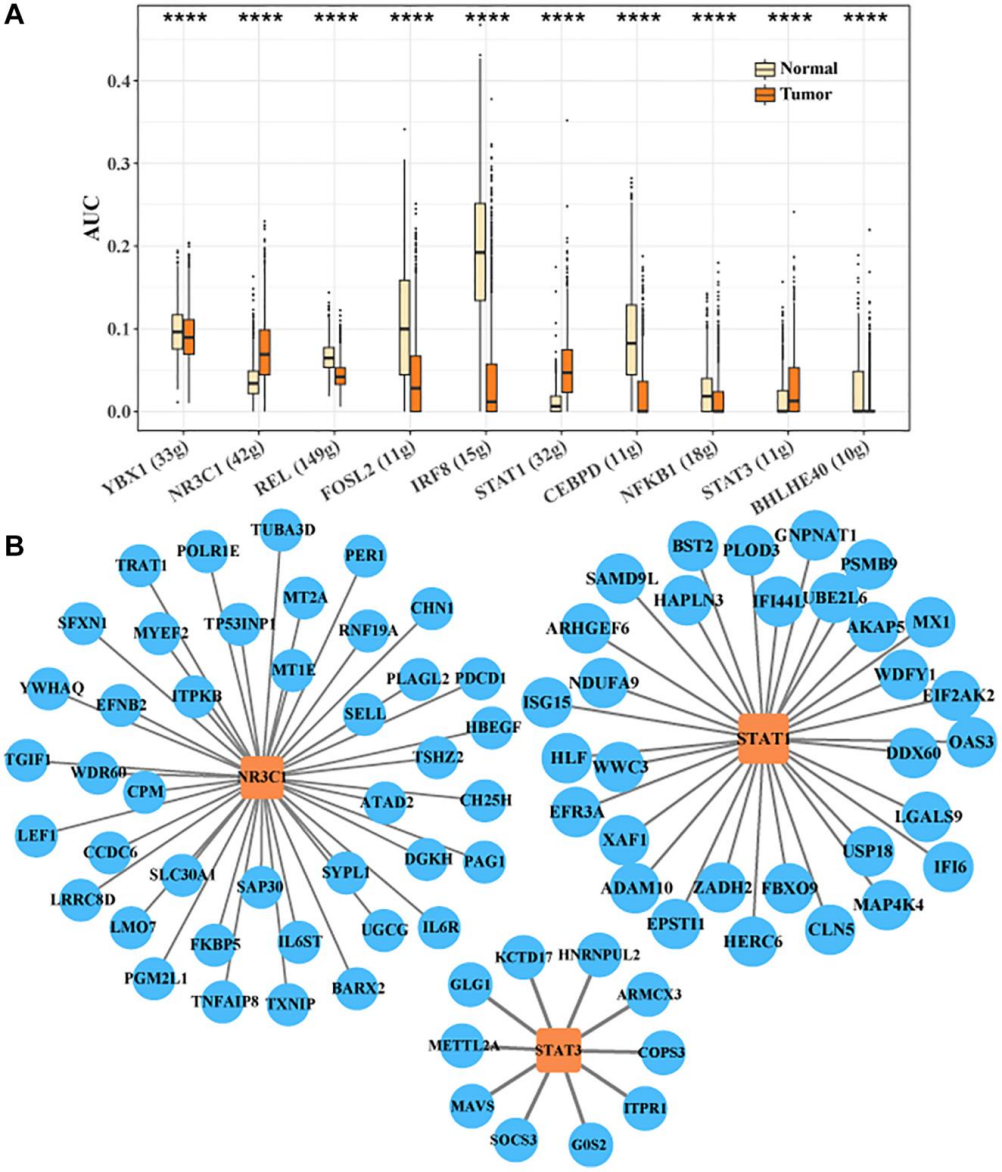
**Transcription factor regulatory network in CTLA4+ T cells.** (**A**) Important transcription factors in CTLA4+ T cells identified by SCENIC analysis. (**B**) Network of target genes regulated by NR3C1, STAT1 and STAT3.

We identified the transcription factor regulatory network in CTLA4+ T cells. Ten key transcription factors were identified, YBX1, NR3C1, REL, FOSL2, IRF8, STAT1, CEBPD, NFKB1, STAT3, BHLHE40. We found that the activities of target genes regulated by NR3C1, STAT1, and STAT3 were higher in tumor tissues, implying that NR3C1, STAT1, and STAT3 played critical regulatory roles for the development of CTLA4+ T cell subsets (Figure 5A). The transcription factor regulatory network of downstream target genes regulated by NR3C1, STAT1 and STAT3 is shown in Figure 5B. Sell activity correlates with overall survival in HCC and may be an adjunctive biomarker for regulatory immunotherapy (PMID: 35702258).

## DISCUSSION

TIICs are important regulators in cancer and are inextricably linked to the outcomes of early response, chemotherapy, and immunotherapy [33, 34]. In this study, we performed a comprehensive data analysis by single-cell sequencing data from HCC samples and normal samples in GSE162616. The landscape of TIICs in HCC was determined by marker genes of immune cells.

According to our observations, T cells and NK cells were the most predominant in the tumor microenvironment of HCC. Three cell subtypes were present in T cells, CTLA4+ T cells, IL1RL1+ T cells, and LAG3+ T cells. CTLA4 was considered as one of the key immune checkpoint genes in effective immunotherapy [35]. CTLA4 was a regulator of the active state of T cells, and when T cells were highly expressing CTLA4, T Cells were in an immunocompromised state [26, 30]. IL1RL1 was also a major impediment to anti-tumor immune responses, and a study by Sun et al. noted that high expression of IL1RL1 signaling in Treg promoted fibrosis and immunosuppression in cancer-associated fibroblasts [36]. Deregulation of the IL1RL1 activation state in TIICs was an effective measure to remodel the anti-tumor response. CD4+ T Cells enrichment was found to be associated with a lower risk of early recurrence [37]. Expression of LAG3, a co-inhibitory receptor for CD4+ T Cells, leads to impeded T cell activation and functional exhaustion [38]. In our study, CTLA4+ T cells, IL1RL1+ T cells, and LAG3+ T cells identified in tumor tissues specifically overexpressed CTLA4, IL1RL1, and LAG3, respectively, which indicated that T Cells in HCC might be in the state of inhibition of activation or depletion of function, and were unable to properly achieve anti-tumor-related killing function. In addition, the results also showed that the abundance of these three T cells in tumor tissues was higher than that in normal liver tissues. These results further suggested that T Cells in HCC might be in an active inhibitory state or functionally depleted state. Liver-resident NK (LrNK) cells and conventional NK (cNK) cells were significantly reduced in HCC, and the T-cell inhibitory receptor Tim-3 was significantly upregulated in both NK cell subtypes, inhibiting their cytokine secretion and cytotoxic activity. This suggests that Tim-3-mediated interference with PI3K/mTORC1 signaling is responsible for the dysfunction of both tumor-infiltrating NK cell subtypes [39]. IL-15-activated NK cells, employing antibodies to promote antibody-dependent cellular cytotoxicity (ADCC), are a novel method of killing tumor cells circumventing tumor immune escape [40]. In HCC the presence of several special T-cell subsets, CD137+ T cells and ICOS+ T cells, presented antigenic activation [41]. The subpopulations of T cells and NK cells identified in this study, likewise showed different functions, enriching the categorization and deepening new insights into the functions of T cells and NK cells in HCC.

In our follow-up study, we found that high CTLA4+ T cells predicted a poor prognosis for HCC. IL1RL1 is normally associated with pro-inflammatory responses, whereas LAG3 (lymphocyte activation gene 3) is an immune checkpoint molecule associated with T-cell depletion and suppression of immune responses [31, 36]. It was found that Amphiregulin couples IL1RL1+ Tregs and cancer-associated fibroblasts to hinder anti-tumor immunity [36]. However, our study only demonstrated T cell subsets expressing different marker genes and did not determine the presence of Tregs. It is likely that it is IL1RL1+ Tregs that play a role in HCC. There is a great deal of disease heterogeneity among HCC patients, and different stages of the disease may be differently dependent on immunomodulatory responses. IL1RL1+ T cells and LAG3+ T cells may play different roles in early or locally progressive HCC, whereas in advanced stages or extensive metastases, their roles may become less significant due to changes in the tumor microenvironment. Overall, our study revealed the presence of functionally depleted CTLA4+ T cells, IL1RL1+ T cells, and LAG3+ T cells in HCC. Among them, CTLA4+ T cells were the cell subpopulation affecting the survival of HCC, and the functions of IL1RL1+ T cells and LAG3+ T cells in HCC need to be further investigated in depth.

Two cell subtypes were found in NK cells, B3GNT7+ NK cells, and FGFBP2+ NK cells. Few studies have explored the function of B3GNT7 in liver cancer and immune cells. The hepatic metastatic and migratory abilities of colon cancer cells were significantly enhanced when B3GNT7 was overexpressed in the cells [42]. This showed that B3GNT7 might be a pro-cancer progression gene. FGFBP2 was the signature gene for cytotoxic killing function in NK cells [27]. In further analysis, HAVCR2 and TGFB1 were highly expressed in FGFBP2+ NK cells. It was found that HAVCR2 was a signature of the end state of NK cells in NK cells, and high expression of HAVCR2 implied functional exhaustion of NK cells [28]. FGFBP2+ NK cells with high expression of HAVCR2 were also a subpopulation of functionally depleted cells in HCC. Impairment of these functional anticancer cells was a potential cause of HCC progression.

Finally, we identified a transcription factor regulatory network centered on NR3C1, STAT1 and STAT3 in CTLA4+ T cells. NR3C1 was the signature gene during differentiation of CD 8+ T cells and regulated the formation of memory precursor cell fates in CD 8+ T cells [43]. NR3C1 formed a transcription factor regulatory network with PDCD1. PDCD1 was also recognized as PD-1. There was a synergistic mechanism between the connection of CTLA-4 and PD-1 to inhibit T cells activation [44]. The TRIB3/STAT1/CXCL10 axis modulated the infiltration abundance of CD 8+ T cells in tumor tissues, leading to the immune escape phenomenon [45]. In another study, STAT3 was also found to be critical for the developmental formation of terminal CD 8+ T cells [46]. Combined with our study, CTLA4+ T cells were a highly abundant and depleted cell subpopulation in HCC, which led to suboptimal survival in HCC patients. NR3C1, STAT1, and STAT3 showed higher expression levels in tumor tissues, and all of these transcription factors were shown to be associated with T cell depletion status and terminal T cells. In summary, CTLA4+ T cells were probably extremely important cell types in HCC, and inhibition of its infiltrative abundance and function could be considered as a novel therapeutic tool.

However, this study still presented limitations. This study was based on data analysis from the GEO and TCGA databases and lacked data from cell or animal experiments. Follow-up experiments are needed to further validate the function of depleted T cell and NK cell subtypes in HCC progression.

## CONCLUSION

Overall, we analyzed single-cell data in tumor tissues and normal tissues of HCC. Our findings demonstrated the presence of exhausted characteristic T cells cell subtypes and NK cells cell subtypes in HCC tissues, which could be essential cell subpopulations in cancer progression. CTLA4+ T cells play a key role in immune escape in HCC, as evidenced by the fact that they are highly expressed in tumor tissues and associated with poor prognosis. Further analyses revealed that CTLA4+ T cells achieve their functions through a specific network of transcription factors, suggesting that by targeting these key transcription factors, we may be able to restore the immune surveillance function of these cells and offer new therapeutic hope for HCC patients.

## Supplementary Materials

Supplementary Figures
