# Supplementary material for: Single-cell data revealed exhaustion of characteristic NK cell subpopulations and T cell subpopulations in hepatocellular carcinoma
Source: Aging (Albany NY). 2024 Apr 5;16(7):6550–65. doi: 10.18632/aging.205723 (PMC11042964; doi:10.18632/aging.205723)
Supplement: Supplementary Figures [file aging-16-205723-s001.pdf]

SUPPLEMENTARY FIGURES

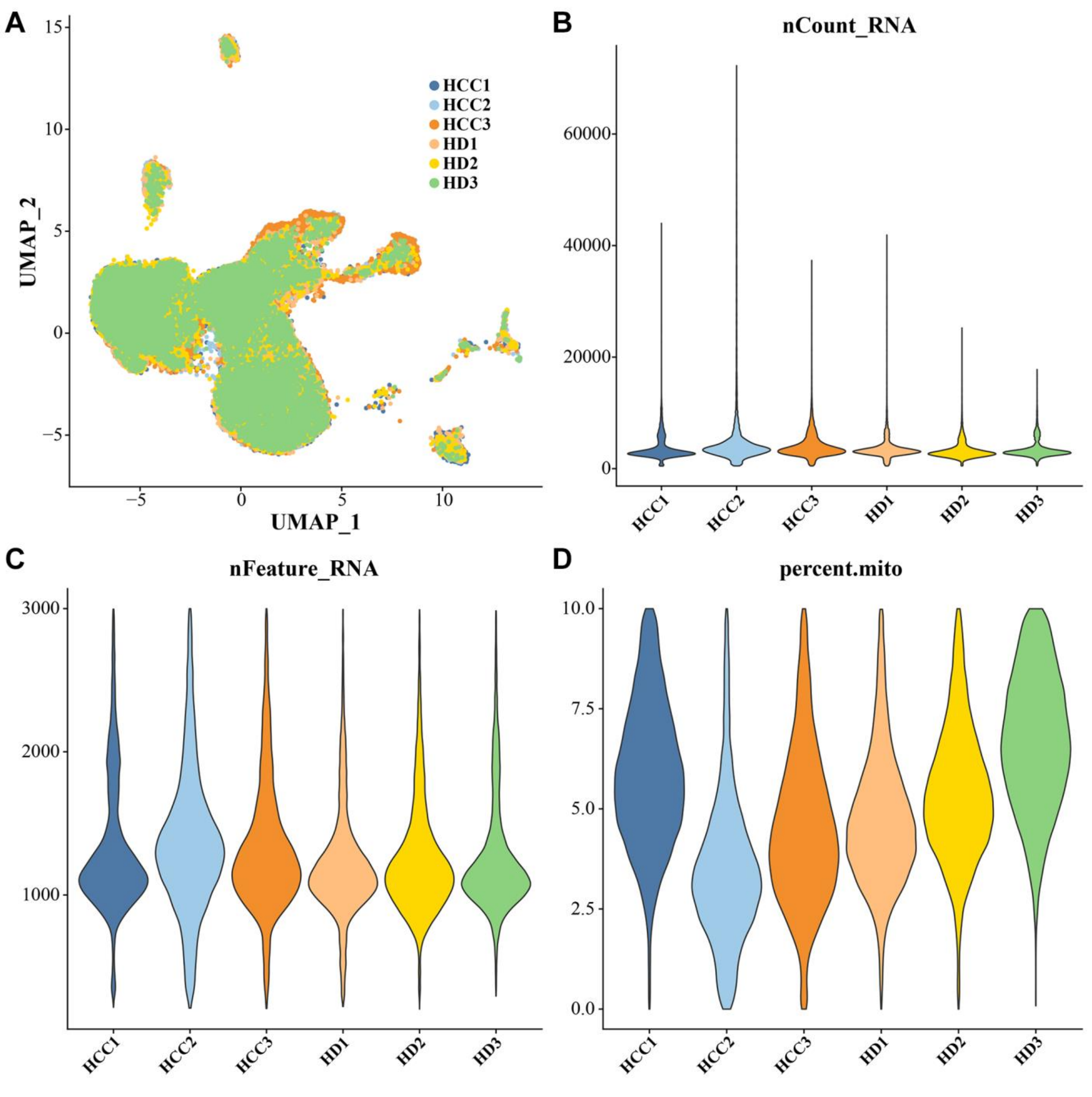

Supplementary Figure 1. scRNA-seq data processing. (**A**) Distribution of cells in six samples after removal of batch effects. (**B**) Distribution of the number of detected UMIs in the samples after filtering. (**C**) Number of detected genes in the samples after filtering. (**D**) Proportion of mitochondrial genes detected in the samples after filtering.

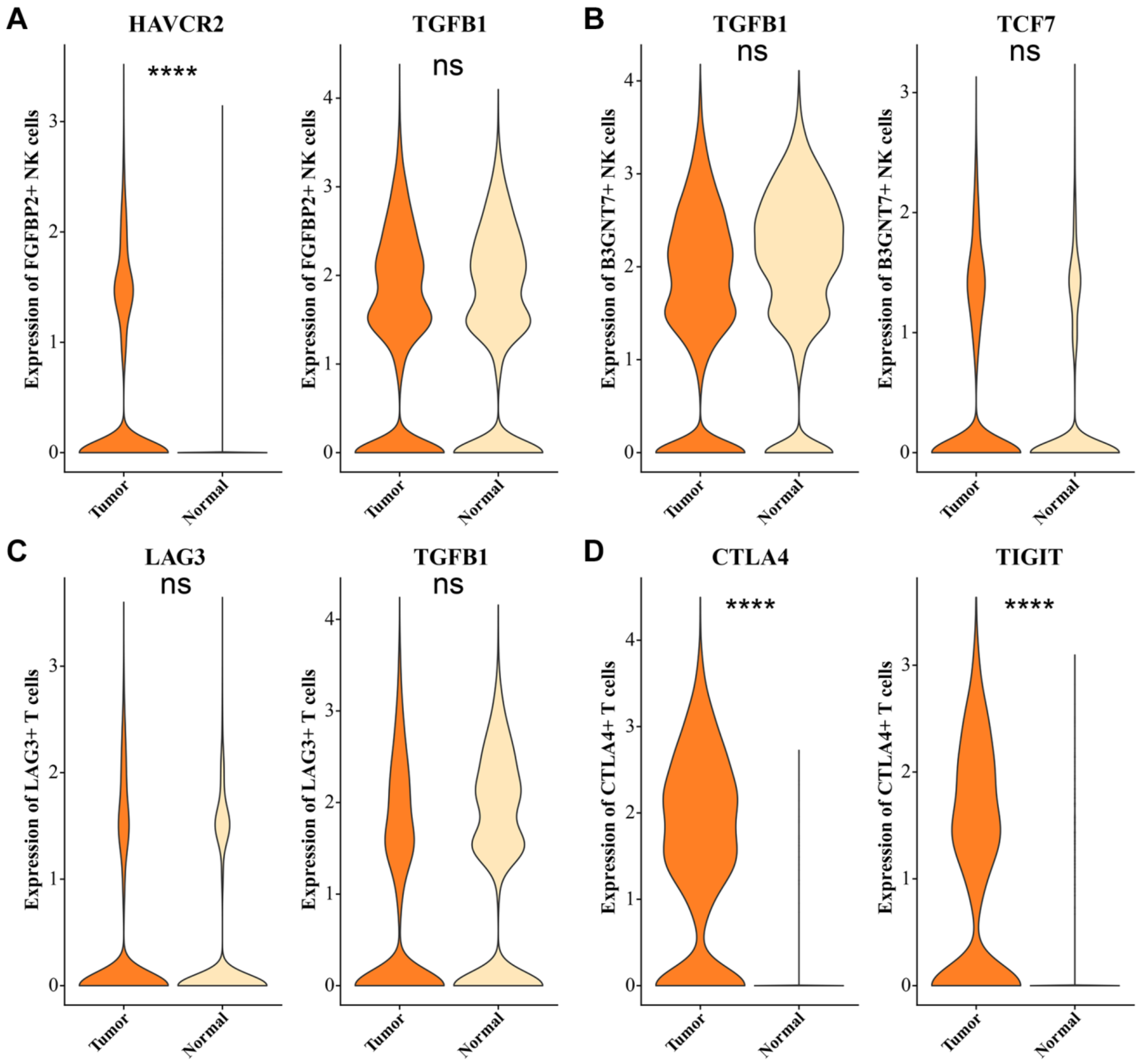

Supplementary Figure 2. Expression levels of marker genes in FGFBP2+ NK cells, B3GNT7+ NK cells, LAG3+ T cells, CTLA4+ T cells. (**A**) Expression levels of HAVCR2 and TGFB1 in FGFBP2+ NK cells. (**B**) Expression levels of TGFB1, TCF7 in B3GNT7+ NK cells. (**C**) Expression levels of LAG3, TGFB1 in LAG3+ T cells. (**D**) Expression levels of CTLA4, TIGIT in CTLA4+ T cells.

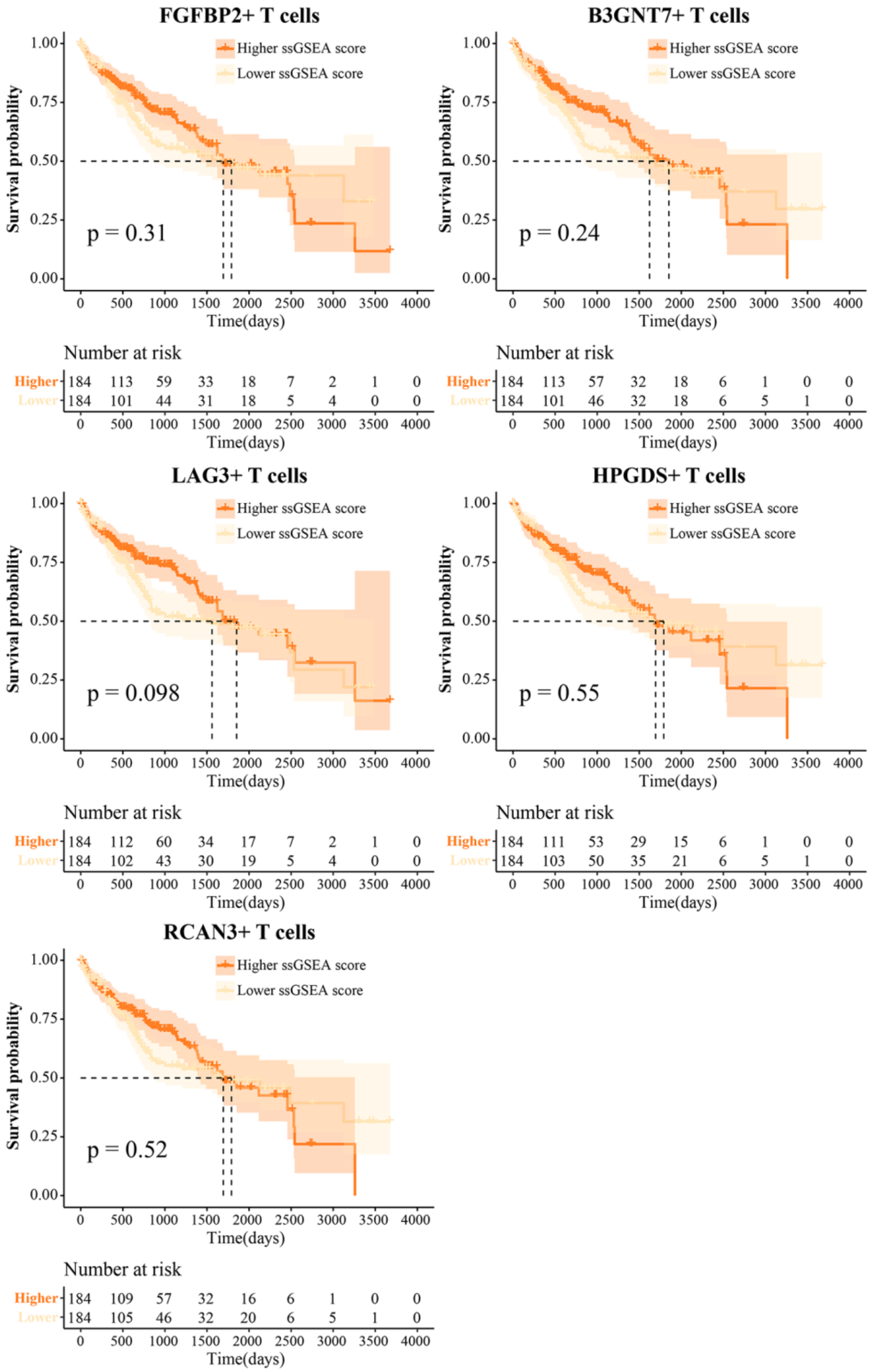

Supplementary Figure 3. K-M curves of HCC patients in the grouping of FGFBP2+ T cells, B3GNT7+ T cells, LAG3+ T cells, HPGDS+ T cells, and RCAN3+ T cells.
